# Mediterranean spotted fever: case series of 24 years (1989–2012)

**DOI:** 10.1186/s40064-015-1042-3

**Published:** 2015-06-17

**Authors:** Pedro Crespo, Diana Seixas, Nuno Marques, Joaquim Oliveira, Saraiva da Cunha, A Meliço-Silvestre

**Affiliations:** Infectious Diseases Unit, Centro Hospitalar e Universitário de Coimbra, Praceta Prof. Mota Pinto, 3000-075 Coimbra, Portugal

**Keywords:** Mediterranean spotted fever, *Rickettsia conorii*, Eschar, Tick-bite

## Abstract

**Purpose:**

Mediterranean spotted fever (MSF) is the most prevalent zoonosis in Portugal. To characterize it’s evolution between 1989 and 2012, the authors reviewed the cases diagnosed at their unit during this period.

**Methods:**

Review of clinical records of patients with MSF diagnosis, between 1989 and 2012.

**Results:**

Data from 250 patients was included, 54% male. Mean age at diagnosis was 58 years (11–92). Mean annual incidence was 10 cases, with clear summer predominance. Most patients, 78% lived in rural areas, 34% had contact with dogs and 10% noticed the tick bite. Most common symptoms were: fever (98%), myo-arthralgia (64%) and headache (48%). Maculopapular rash was noticed in 87%, affecting palms in 77% and soles in 69%. Inoculation eschar was found in 60%, mostly located on the trunk. Treatment included doxycycline in 86% and chloramphenicol in 12%, with a mean duration of 8 days. Most frequent blood test abnormalities were C-reactive protein, lactate dehydrogenase, aspartate aminotransferase and alanine transaminase elevations and thrombocytopenia. First serologic evaluation was positive in 37% (78/212), having seroconversion been documented in 85% (72/85). Most frequent complication was acute renal injury. ICU admission occurred in 5%. Average length of hospital stay was 11.2 days (1–106), with a mortality of 3.6%.

**Conclusions:**

In our series, there was clear summer predominance of MSF, which had rural origin in 78%. Most common symptoms were fever, myo-arthralgia and headache. Maculopapular rash was noticed in 87% of cases and inoculation eschar in 60%. Most cases had favourable outcome, having 5% been admitted to ICU. Mortality was 3.6%.

**Electronic supplementary material:**

The online version of this article (doi:10.1186/s40064-015-1042-3) contains supplementary material, which is available to authorized users.

## Background

Mediterranean spotted fever (MSF) is a zoonosis endemic in the Mediterranean basin. Conor and Bruch first described it in Tunisia in 1910 as a disease that caused high fever and spots. It was soon reported in other regions around the Mediterranean (Walker and Raoult 2010; Rovery et al. 2008; Rovery and Raoult 2008). In Portugal, Delfim Pinheiro made the description of the first cases in 1923 and in 1930, Ricardo Jorge’s work contributed to the divulgation of the disease, naming it “febre escaro-nodular” (fever with eschar and nodules), name by which it is still known nowadays (Louro et al. 2006; Sousa et al. 2003).

MSF is an emerging/reemerging disease in some countries in which it’s incidence has varied in a cyclic manner (Rovery et al. 2008; Rovery and Raoult 2008; Oliveira and Côrte-Real 1999). Though its incidence in Portugal has decreased throughout the last decade, MSF is still the most prevalent zoonosis, with 140 cases notified in 2010 (1024 cases in 1996) (Santos 2014). This result is probably biased by sub-notification. Although of mandatory notification in Portugal, it is estimated that only 14% of MSF cases are notified (Sousa et al. 2003).

The causative agent is *Rickettsia conorii*, an obligate intracellular and extremely fastidious Gram-negative bacterium (Oliveira and Côrte-Real 1999; Galvão et al. 2005). In Portugal, there are two strains of the *Rickettsia conorii* complex, the *Malish* and the *Israeli tick typhus* strain (Sousa et al. 2003, 2008). The main vector of the disease is *Rhipicephalus sanguineus*, the brown dog tick (Rovery et al. 2008; Rovery and Raoult 2008; Oliveira and Côrte-Real 1999; Figueira-Coelho et al. 2010; Duque et al. 2012). The tick itself may acts as a reservoir for *Rickettsia,* as these bacteria can be maintained in ticks through transtadial and transovarial transmission (Rovery et al. 2008; Sousa et al. 2003; Duque et al. 2012; Sociedade Portuguesa de Pediatria 2005; Parola et al. 2013; Brouqui et al. 2004). Other reservoirs are thought to be dogs, rabbits and some small rodents (Rovery et al. 2008; Rovery and Raoult 2008; Sociedade Portuguesa de Pediatria 2005), but there is no consensus in this point. Humans are an accidental host of Rickettsia and play no role in maintaining this bacterium in nature (Rovery and Raoult 2008; Galvão et al. 2005; Parola et al. 2013).

Although in some countries men are more affected than women (Rovery and Raoult 2008), gender distribution in Portugal is homogeneous (Sousa et al. 2003). Although it can affect individuals of all ages, in our country, the highest incidence rate is observed in children (1–4 years old). However, it is in the elderly patient and in people with comorbidities (diabetes mellitus, immunodepression, cardiac pathology, chronic alcoholism and glucose-6-phosphate dehydrogenase deficiency) that the disease has higher complication and mortality rates (Sousa et al. 2003; Oliveira and Côrte-Real 1999; Parola et al. 2013).

The disease incidence has a seasonal variation, related with the tick activity, being more prevalent in hotter months, especially May through September. Yet still, cases have been reported throughout the whole year (Rovery and Raoult 2008; Oliveira and Côrte-Real 1999).

In Portugal, cases have been described in all districts, being more frequent in those of Bragança, Beja and Coimbra (Sousa et al. 2003).

After an asymptomatic incubation period (one week), the onset of disease is usually abrupt (Rovery and Raoult 2008). The classic clinical triad is composed of high fever (>39°C), maculopapular non-pruriginous rash with palmo-plantar involvement and an inoculation eschar at the site of the tick bite (Walker and Raoult 2010; Rovery and Raoult 2008; Sousa et al. 2003; Oliveira and Côrte-Real 1999). Flu-like symptoms are usually also present (Walker and Raoult 2010; Oliveira and Côrte-Real 1999; Parola et al. 2013). Faccini-Martínez et al. (2014) suggest MSF inclusion in the group of exanthematic rickettsioses syndrome with a probability of inoculation eschar and maculopapular/purpuric rash.

The pathogenic basis of the disease is a systemic vasculitis (La Scola and Raoult 1997), being the eschar the gateway of *Rickettsia* into the host organism and probably the first reaction of the organism to control the infection (Parola et al. 2013). It can be difficult to identify (Rovery et al. 2008) and may even be absent in 14–40% of cases (Rovery and Raoult 2008), as it is common in infection by *Rickettsia conorii* subsp. *israelensis* (Sousa et al. 2003, 2008; Parola et al. 2013; Bacellar et al. 1999). The description of cases with multiple eschars may be linked to the increasing aggressiveness of *Rhipicephalus sanguineus*, eventually related with climatic changes (Rovery and Raoult 2008; Parola et al. 2013), but may also be related with other Rickettsiosis, such as lymphangitis associated rickettsioses (LAR), caused by *Rickettsia sibirica* (Fournier et al. 2005; Sousa et al. 2006; Pereira et al. 2011).

The diagnosis is based on clinical, epidemiological and laboratorial criteria (Oliveira and Côrte-Real 1999; Sociedade Portuguesa de Pediatria 2005). In Portugal, it is recommended that the suspected cases have a laboratorial confirmation (Sousa et al. 2003; Oliveira and Côrte-Real 1999).

Complete blood count abnormalities are non-specific. Leukocytosis, leukopenia or normal white blood cell count may be present. Thrombocytopenia is frequent. Inflammatory markers (sedimentation rate or C-reactive protein) and liver function tests are usually elevated (Oliveira and Côrte-Real 1999; Brouqui et al. 2004; Raoult et al. 1986).

Direct identification of *Rickettsia* by cellular culture, immune-histological techniques or PCR DNA amplification test from skin or eschar biopsies is the most specific method of diagnosis (Walker and Raoult 2010; Sousa et al. 2003). Recently, PCR from eschar swabs has been described as being equally useful in identification of *Rickettsia* (Parola et al. 2013). The use of PCR allowed for the discovery of new species of Rickettsia, some of which could have been the cause of previously described atypical MSF cases, challenging classic taxonomy (Walker and Raoult 2010; Rovery and Raoult 2008; Bacellar et al. 1999; Sousa et al. 2006, 2013; Pereira et al. 2011; Raoult et al. 1997).

As direct identification of *Rickettsia* is expensive, and usually restricted to reference laboratories, serological tests are still the most widely available, easy to perform and most frequently used diagnostic method (Oliveira and Côrte-Real 1999; Parola et al. 2013). Weil–Felix reaction is obsolete and not used anymore (Sousa et al. 2003; Oliveira and Côrte-Real 1999; Galvão et al. 2005), being immunofluorescence considered the gold standard by the WHO. The diagnosis is confirmed by seroconversion or by a fourfold rise in titers between acute and convalescence serum samples (Oliveira and Côrte-Real 1999; Sociedade Portuguesa de Pediatria 2005; Brouqui et al. 2004; La Scola and Raoult 1997). Notwithstanding this fact, the selection of antigens used in this method is limited and cross-reacts with different *Rickettsia*, making it difficult to identify the definitive etiological agent (Brouqui et al. 2004; Pereira et al. 2011).

Keeping in mind that early and appropriate antibiotic prescription is crucial for a favourable outcome (Rovery and Raoult 2008; Amaro et al. 2003), clinical evaluation is still the fastest and most precious diagnostic weapon (Galvão et al. 2005). A diagnostic scoring system has been developed to help in MSF diagnosis, based in epidemiologic, clinical and laboratory data (Brouqui et al. 2004; Cascio et al. 2002).

The treatment of MSF is based on antibiotics with good intracellular activity, being doxycycline, 100 mg 2id, the preferred (Rovery and Raoult 2008; Parola et al. 2013; Faccini-Martínez et al. 2014), even in pregnant women (severe cases) and children less than 8 years-old (Faccini-Martínez et al. 2014), as the risk for dental staining is negligible when a single and short course of therapy is used (Rovery and Raoult 2008). Alternatives are macrolides, particularly interesting in pregnant women (Parola et al. 2013; Faccini-Martínez et al. 2014; Cascio et al. 2002; Colomba et al. 2006) and chloramphenicol (Oliveira and Côrte-Real 1999; Faccini-Martínez et al. 2014). Usefulness of fluoroquinolones has been questioned by recent studies (Parola et al. 2013; Faccini-Martínez et al. 2014; Botelho-Nevers et al. 2011). A response-guided therapy is advised, keeping antibiotic therapy until 24–48 h after fever defervescence (Rovery and Raoult 2008; Sociedade Portuguesa de Pediatria 2005).

Clinical improvement usually occurs within 48 h of treatment initiation and patients usually recover within 10 days with no sequelae. Although reinfection can occur in some patients, MSF is always an acute disease (Rovery and Raoult 2008).

Although usually benign, life-threatening cases may also occur, with sepsis, shock and multiple organ failure (Rovery and Raoult 2008; Oliveira and Côrte-Real 1999; Sousa et al. 2008; Figueira-Coelho et al. 2010; Duque et al. 2012; Parola et al. 2013; Amaro et al. 2003; Colomba et al. 2014).

As the vector and reservoirs for *Rickettsia conorii* move freely in nature, it is impossible to eradicate it (Sousa et al. 2003; Sociedade Portuguesa de Pediatria 2005). Therefore, it is important to take measures to prevent infection, such as: avoiding tick infested areas, use of protective clothing and repellent, thorough search for ticks after possible environmental exposure or deworming of domestic reservoir animals (Oliveira and Côrte-Real 1999; Sociedade Portuguesa de Pediatria 2005; Garcia-Alvarez et al. 2013).

In order to better characterize it’s evolution between 1989 and 2012, we reviewed the cases followed-up in the authors unit during this period.

## Methods

The authors made a retrospective review of the files of the patients followed-up for MSF (as defined by the clinical team that cared for the patient), between 1st January 1989 and 31st December 2012. MSF diagnostic was based on clinical, epidemiological and laboratorial aspects. Serologic confirmation was considered when one of the following criteria was met: IgG >128 in acute phase test, seroconversion or fourfold titer rise between acute and convalescent test. In the cases in which this did not happen, clinical evaluation was considered the most important diagnostic criteria.

Informed consent was obtained from the patients and their data was kept anonymous.

## Results

### Epidemiology

Data from 252 patients was collected, 250 were included (see atypical presentation in "Results"), 54% (n = 134) male, with a male to female ratio of 1:0.87. Mean age at diagnosis was 58 years (11–92). Age distribution is shown in Additional file 1: Figure S1.

The mean annual incidence was 10 cases, varying from 1 case in 1997 to 21 in 2004. Since then it was apparent a slight decrease in the number of cases diagnosed each year (Additional file 2: Figure S2). Clear summer predominance was observed (Additional file 3: Figure S3), with 78% of the cases occurring between July and September (n = 196). Despite this fact, there have been cases diagnosed in the months of February, November and December.

The most frequent comorbidities presented by the patients are shown in Additional file 4: Table S1.

The majority of patients (78%, n = 194) lived in rural areas. Contact with dogs was referred by 34% (n = 85) and only 10% (n = 26) noticed a tick bite.

### Clinical presentation

At admission, patients reported in average 5.6 days (1–30) of evolution of symptoms.

The clinical manifestations are described in Additional file 5: Table S2. The most common symptoms were: fever (98%, n = 245), myalgia and arthralgia (64%, n = 159), headache (48%, n = 119) and asthenia (27%, n = 68).

On physical examination, maculopapular rash was noticed in 87% of patients (n = 218), affecting the palms in 77% (n = 168) and the soles in 69% (n = 150). The inoculation eschar (*tache noire*) was found in 60% (n = 151), being located on the trunk in 58% (87/151), lower limbs in 28% (42/151) and upper limbs in 11% of cases (16/151) (Additional file 6: Table S3). Multiple eschars were found in just 2% (3/151). Enlarged lymph nodes in the drainage area were present in 13% (19/151) of those with inoculation eschar.

The characteristic rash was absent in 13% (n = 32) of patients. Of these, 34% (11/32) had fever and an inoculation eschar.

The classic triad of fever, maculopapular rash and inoculation eschar was present in 49% (123/250) of patients.

In patients with available data (n = 54), rash followed fever in 3.2 days (0–11).

### Atypical presentations

The two cases excluded were considered probable cases of atypical rickettsioses and not atypical presentations of MSF. One of the cases was a patient who presented with flu-like symptoms, two eschars and lymphangitis from one of them to the draining node. The other was a 38 year-old woman, who sought medical care after having noticed an eschar in the head. Painful cervical lymph node enlargement was noticed, but she presented no other complaints. Both cases were treated with doxycycline with complete recovery.

Throughout the period of the study, the eschar was detected in the head in only 3% (4/151) of cases. Another case, a 58 year-old male, also presented an inoculation eschar on the head, but presented the classical clinical triad of MSF in the month of June 2012. Culture and PCR DNA amplification of *Rickettsia* were not attempted in the last two cases.

One of the cases diagnosed in December, was a 33-year-old male veterinarian, who noticed a tick-bite on his leg. He presented with fever, myalgia, headache and an inoculation eschar on the right leg, with enlarged inguinal lymph nodes. No rash was noticed. Serologic test was positive both in the acute and convalescence phase, with a eightfold increase in titter (from 160 to 1280). He was given doxycycline and had a favourable outcome.

### Laboratory results

In what regards complete blood cell count evaluation, 10% (25/240) presented leukopenia (<4 G/L) and 19% (46/240) leucocytosis (>10 G/L). Thrombocytopenia (<150 G/L) was detected in 60% (151/250) and thrombocytosis (>400 G/L) in only 2% (5/250).

The most frequent biochemistry panel abnormalities were C-reactive protein (98%, 229/234), lactate dehydrogenase (LDH—86%, 206/240), aspartate aminotransferase (AST—74%, 184/249), alanine transaminase (ALT—54%, 135/250) and gamma-glutamyl transpeptidase (gamma-GT—47%, 118/250) elevations.

The acute phase serology (immunofluorescence assay—taken at admission) was done in 85% (n = 212) of cases and was positive in 37% (78/212). Seroconversion was documented in 85% (72/85). In 62% (131/212) of patients, paired serologies were obtained. Convalescent phase serologies were done 6–8 weeks after the acute phase ones. In 14% (n = 35) of patients, no serology test was done. The results of serologic evaluations are described in Additional file 7: Table S4. In this table, although the titers values begin in 1/80, only titers above 1/128 were considered diagnostic. In the authors hospital only IgG titers were made available. Serologic tests for other pathogens were also done, but their results were not collected.

Identification of *Rickettsia* subspecies was tried in 21 cases, by culture and PCR amplification (tests done in the National Institute of Health). In two cases it was identified the *Malish* strain of *Rickettsia conorii* and in two other, the *Israeli spotted fever* strain (all four had serologic seroconversion documented). No identification was possible in the 17 remaining cases.

### Treatment and outcome

The treatment included doxycycline in 86% (n = 215) and chloramphenicol in 12% (n = 30), with a mean duration of 8 days. Azithromycin was only used once, in a 14-year-old boy, with resolution of disease.

The most frequent complication was acute renal injury, observed in 16% (n = 40) of cases and respiratory failure, in 15% (n = 37). Multiple organ failure justified admission to intensive care unit in 5% (n = 13) of cases. Rare but severe complications included two cases of necrosis of the extremities (one case of two toes and another of three fingers of one hand) and one case of extended skin necrosis of the leg.

The average length of hospital stay was 11.2 days (1–106), with a mortality of 3.6% (n = 9). In the latter cases, all patients had comorbidities (chronic alcoholism, heart failure and COPD) and were over 60-years old.

## Discussion

Being MSF the most prevalent zoonosis in Portugal, we wanted to characterize the patients followed in our unit and evaluate if in the study period there were some changes in epidemiological data and clinical presentation.

Gender balance was similar to previous reports from our country (Sousa et al. 2003), although different from some studies in which a clear male predominance was found (Rovery and Raoult 2008).

Age distribution was the expected, as the author’s unit follows mainly adult cases.

The cyclical variation of the incidence throughout the study period was also found in other series, and is justified by the emergent or re-emergent characteristic of MSF (Rovery and Raoult 2008; Sousa et al. 2003).

The summer predominance that we found (78% of the cases occurring between July and September), was expected, and described in many articles (Rovery and Raoult 2008; Sousa et al. 2003; Oliveira and Côrte-Real 1999). The existence of cases diagnosed in colder months, as the one described with admission in December, testifies that *R. sanguineus* preserves activity throughout the year (Rovery and Raoult 2008; Oliveira and Côrte-Real 1999), even more in Portugal, a country with warm temperatures even in the colder months.

From the five most prevalent comorbidities, at least three are usually considered risk factors for severe MSF: diabetes, heart failure and chronic alcoholism (Sousa et al. 2003; Oliveira and Côrte-Real 1999; Sousa et al. 2008; Sociedade Portuguesa de Pediatria 2005). Yet still, only 5% of patients (n = 13) needed intensive care unit admission.

The clear predominance of rural origin (78%, n = 194) of the affected patients found in this series contrasts with recent data that state that most of the cases are either of urban origin (Rovery and Raoult 2008) or that urban and rural populations are equally affected (Parola et al. 2013). This difference is easily justifiable by the geographic location of our centre, and its vicinity with rural areas. The contact with dogs was only described in 34% (n = 85) of our patients, what calls into question the role of dogs as a reservoir of *Rickettsia conorii*. Although contact with other animals (small rodents, cats or farm animals) was found in 14 cases, the fact that *Rhipicephalus sanguineus* itself acts not only as vector, but also as reservoir (Rovery et al. 2008; Sousa et al. 2003; Duque et al. 2012; Sociedade Portuguesa de Pediatria 2005; Parola et al. 2013; Brouqui et al. 2004), may justify many of the cases without clear relation with dogs or other animals. Despite this fact, the true reservoir of *Rickettsia conorii* is still unknown (Rovery et al. 2008). In our series, tick bite was noticed only by 10% (n = 26) of patients. Although inferior to other Portuguese case series (Sousa et al. 2008), this result is easily understood by the difficulty to find a tick attached to the patient’s body, as it’s size is very small, particularly in the nymph stages. This stage of ticks has a peak activity in the warmer months (Rovery and Raoult 2008), exactly the ones in which more cases have been diagnosed.

The most frequent clinical manifestations found in our series were fever, rash, myoarthralgia, inoculation eschar and headache. These manifestations are similar with the classic descriptions of MSF. Despite this fact, the relative prevalence is different from other national series. Rita de Sousa et al. (2008), described a series with higher prevalence of inoculation eschar, rash, headache and gastrointestinal symptoms, what can be justified by different geographical origin of patients, but most of all, by the severity of cases presented in that series, with almost 30% admitted to an ICU (only 5% in our series).

The presence of an inoculation eschar, varies widely among different series. In ours it was found in 60% (n = 151), a result that is within values presented in other series (Louro et al. 2006; Oliveira and Côrte-Real 1999; Sousa et al. 2008), which varied from 39 to 88%. In our series, in 84% (127/151) of the cases, the inoculation eschar was located on non-exposed areas of skin. It was multiple in only 2% of patients and located on the head in 3%. Although weakly represented in our series, these two characteristics are described in association with different types of Rickettsiosis [multiple eschars with LAR (Faccini-Martínez et al. 2014; Fournier et al. 2005; Sousa et al. 2006; Pereira et al. 2011)—and scalp eschar with “scalp eschar and neck lymphadenopathy following tick bite”—SENLAT (Parola et al. 2013; Faccini-Martínez et al. 2014; Raoult et al. 1997; Sousa et al. 2013; Colomba et al. 2006; Angelakis et al. 2010)]. SENLAT is also know as TIBOLA (tick-borne lymphadenopathy) and DEBONEL (*Dermacentor*-borne necrosis-erythema–lymphadenopathy), and can be caused by agents other than *Rickettsia* (Angelakis et al. 2010).

Although MSF, LAR and SENLAT are all included in the Rickettsiosis syndrome with a probability of inoculation eschar (Faccini-Martínez et al. 2014), they constitute individual clinical entities.

Some clinical presentations previously considered atypical in MSF, such as multiple eschars, lymphangitis, head eschars or cases in colder months, may also be associated to other Rickettsiosis. In the period of the present series, some cases of less common Rickettsiosis have been described by our unit (Pereira et al. 2011; Sousa et al. 2013).

The clinical case excluded from our series, of a patient presenting with two eschars and lymphangitis, was probably considered MSF because of fever, flu-like symptoms and the presence of inoculation eschars. Although not confirmed by laboratory tests, this case fits the description of LAR (Faccini-Martínez et al. 2014; Fournier et al. 2005; Sousa et al. 2006; Pereira et al. 2011), and therefore was excluded from this case series. Also, the case of the patient presenting with an inoculation eschar on the head and an enlarged painful cervical lymph node, without fever or rash, is similar to other cases of SENLAT described in the literature (Parola et al. 2013; Faccini-Martínez et al. 2014; Raoult et al. 1997; Sousa et al. 2013; Colomba et al. 2006) and was also excluded. This diagnostic of MSF in this case was probably assumed because of the identification of a tick-bite and the inoculation eschar. On the other hand, the case diagnosed in December 2009, of a 33 year-old male veterinarian, although not in the predominant season for MSF, presented classic clinical manifestations, associated with serologic results. Using the scoring system described by Brouqui et al. (2004), this case would have 37 points, confirming the diagnostic of MSF. This case stresses the importance of keeping MSF in mind even apart from summer months, particularly in warmer countries.

Clinical and epidemiologic aspects are crucial when diagnosing MSF, as laboratory results take too long to guide therapy, which must be started as early as possible. When patients present characteristic clinical manifestations, in the appropriate season of the year, diagnosis may be straightforward (Sousa et al. 2003; Oliveira and Côrte-Real 1999). On the other hand, atypical cases must trigger the interest of clinicians to try to identify “non-conorii rickettsiosis”, as they can suggest the emergence of Rikettsia species previously unknow in a region. In this process, PCR amplification from collected samples plays a central role, as it allows the identification of the species of *Rickettsia* involved (Rovery and Raoult 2008; Sousa et al. 2008; Parola et al. 2013; Faccini-Martínez et al. 2014). This identification would also enrich epidemiological knowledge about species of *Rickettsia* present in a region (La Scola and Raoult 1997).

Complete blood count evaluation showed thrombocytopenia in 60% (n = 151) of patients, with no major change in leukocyte count. This result is characteristic of MSF (Oliveira and Côrte-Real 1999; Raoult et al. 1986), and resolves with therapy. Biochemistry panel changes were also the usual in this pathology (Oliveira and Côrte-Real 1999; Raoult et al. 1986), with elevation of liver function tests and C-reactive protein.

Acute phase serology (by immunofluorescence assay) was positive in 37% (78/212) of the cases in which it was done. From these, 55% (43/78) presented IgG > 1/128. Seroconversion was documented in 85% (72/85). Paired serologies were obtained in 62% (131/212) of patients. In these, 20% (26/131) had a fourfold increase in titer between acute and convalescent phase. Gathering the serologic results, diagnostic criteria (IgG > 128 in acute phase test, seroconversion or a fourfold titer raise between acute and convalescent test (Oliveira and Côrte-Real 1999; Sousa et al. 2008; Brouqui et al. 2004)—excluding the ones that had already an acute test >128) were met in 63% (133/212) of the patients with serologic evaluation done. These results confirm that clinical evaluation is fundamental to guide diagnostic and treatment, even if later serologic confirmation is obtained.

The time interval between acute and convalescent phase serologies was usually 6–8 weeks, as this was the time when follow-up appointments were usually scheduled.

Subspecies identification, by culture or PCR amplification tests was only successful in four cases, a number that does not allow for any comparison between the diseases caused by these two subspecies. In all this four cases, first serology was negative, being seroconversion confirmed.

The treatment of MSF is based in antibiotics with good intracellular activity, being doxycycline the most used in first-line therapy (Rovery and Raoult 2008; Parola et al. 2013). Alternatives are macrolides, particularly useful in pregnant women and chloramphenicol (Oliveira and Côrte-Real 1999; Parola et al. 2013; Faccini-Martínez et al. 2014; Cascio et al. 2002; Colomba et al. 2006). In our series antibiotic treatment included doxycycline in 86% (n = 215) and chloramphenicol in 12% (n = 30), with a mean duration of 8 days. Azithromycin was only used once, in a 14 year-old boy, with resolution of disease.

Clinical improvement usually occurs within 48 h of treatment initiation and patients usually recover within 10 days with no sequelae. Cases complicated by sepsis, shock and multiple organ failure (Rovery and Raoult 2008; Oliveira and Côrte-Real 1999; Sousa et al. 2008; Figueira-Coelho et al. 2010; Duque et al. 2012; Parola et al. 2013; Amaro et al. 2003; Colomba et al. 2014) are rare, but may occur. They are usually seen in older patients, in people with comorbidities (diabetes mellitus, immunodepression, cardiac pathology, chronic alcoholism and glucose-6-phosphate dehydrogenase deficiency) and in those who had inappropriate or delayed antibiotic treatment (Sousa et al. 2003; Oliveira and Côrte-Real 1999; Parola et al. 2013).

In our series, acute renal injury was the most frequent complication and was observed in 16% (n = 40) of cases, followed by respiratory failure in 15% (n = 37). Although meningoencephalitis is a rare complication, it was considered present in 7% (n = 17) of patients and documented in one case (Duque et al. 2012). Multiple organ failure justified admission to intensive care unit in 5% (n = 13) of cases.

The average length of hospital stay was 11.2 days (1–106), with a mortality of 3.6% (n = 9). The mortality is within Portuguese results presented in other series (Sousa et al. 2003; Oliveira and Côrte-Real 1999; Galvão et al. 2005; Sousa et al. 2008; Amaro et al. 2003). Yet still it decrease from 6% in the 1989–2001 period to 2% in the 2002–2012 interval.

As expected, all the deceased had comorbidities (chronic alcoholism, heart failure and COPD) and were over 60 years old.

The patients that recovered from MSF had favourable outcome, with no sequelae.

## Additional files

Additional file 1:
** Figure S1.** Age distribution. Additional file 2: 
**Figure S2.** Annual incidence. Additional file 3:
** Figure S3.** Monthly distribution. Additional file 4:
** Table S1.** Most frequent comorbidities. Additional file 5:
** Table S2.** Clinical manifestations. Additional file 6:
** Table S3.** Inoculation eschar location. Additional file 7:
**Table S4.** Serologic evaluation results (immunofluorescence assay).
