# Supplementary figures and images for: Mediterranean spotted fever: case series of 24 years (1989–2012)
Source: Springerplus. 2015 Jun 17;4:272. doi: 10.1186/s40064-015-1042-3 (PMC4469589; doi:10.1186/s40064-015-1042-3)

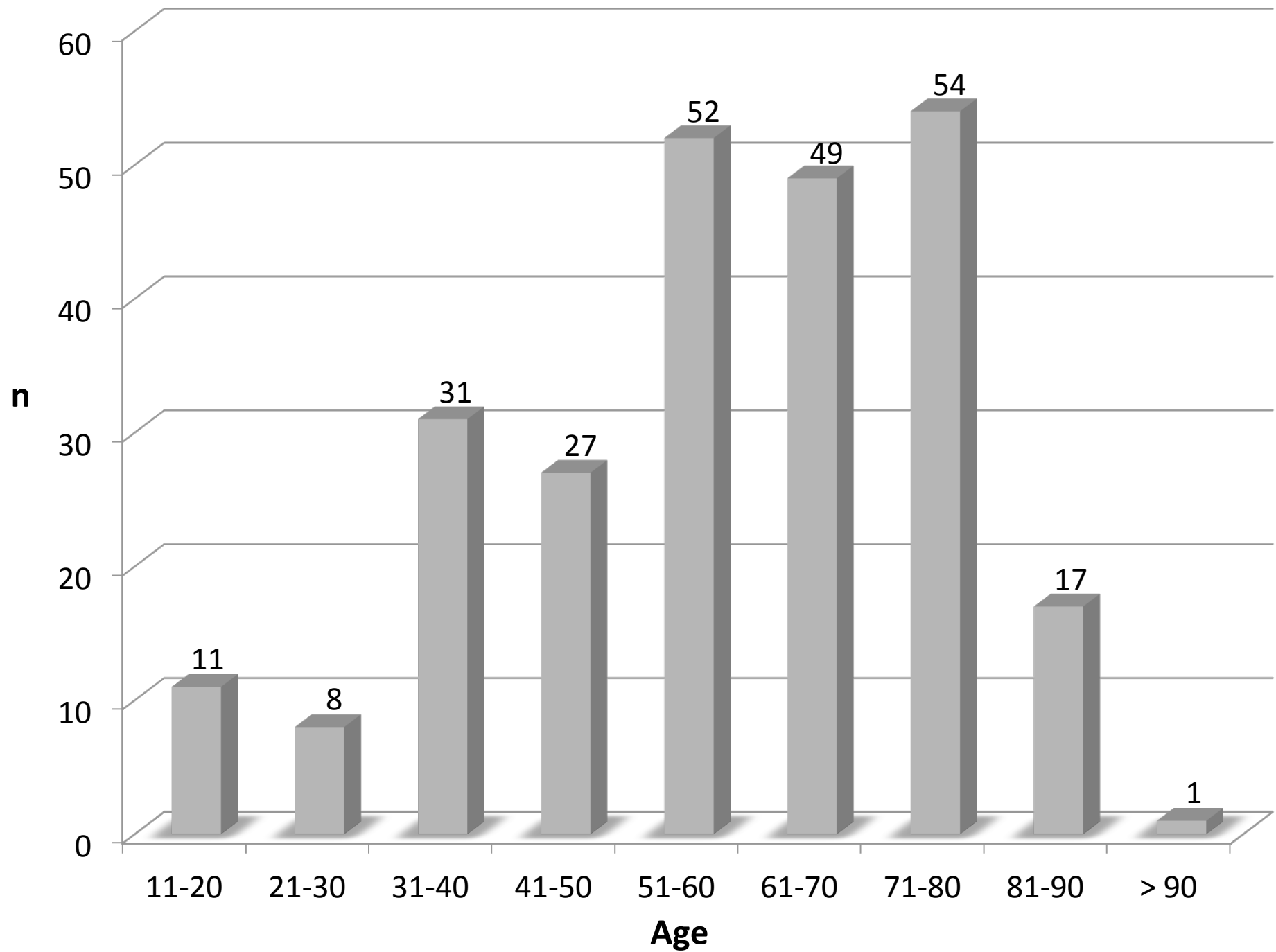

Supplement: Additional file 1: — Figure S1. Age distribution. [file 40064_2015_1042_MOESM1_ESM.pdf]

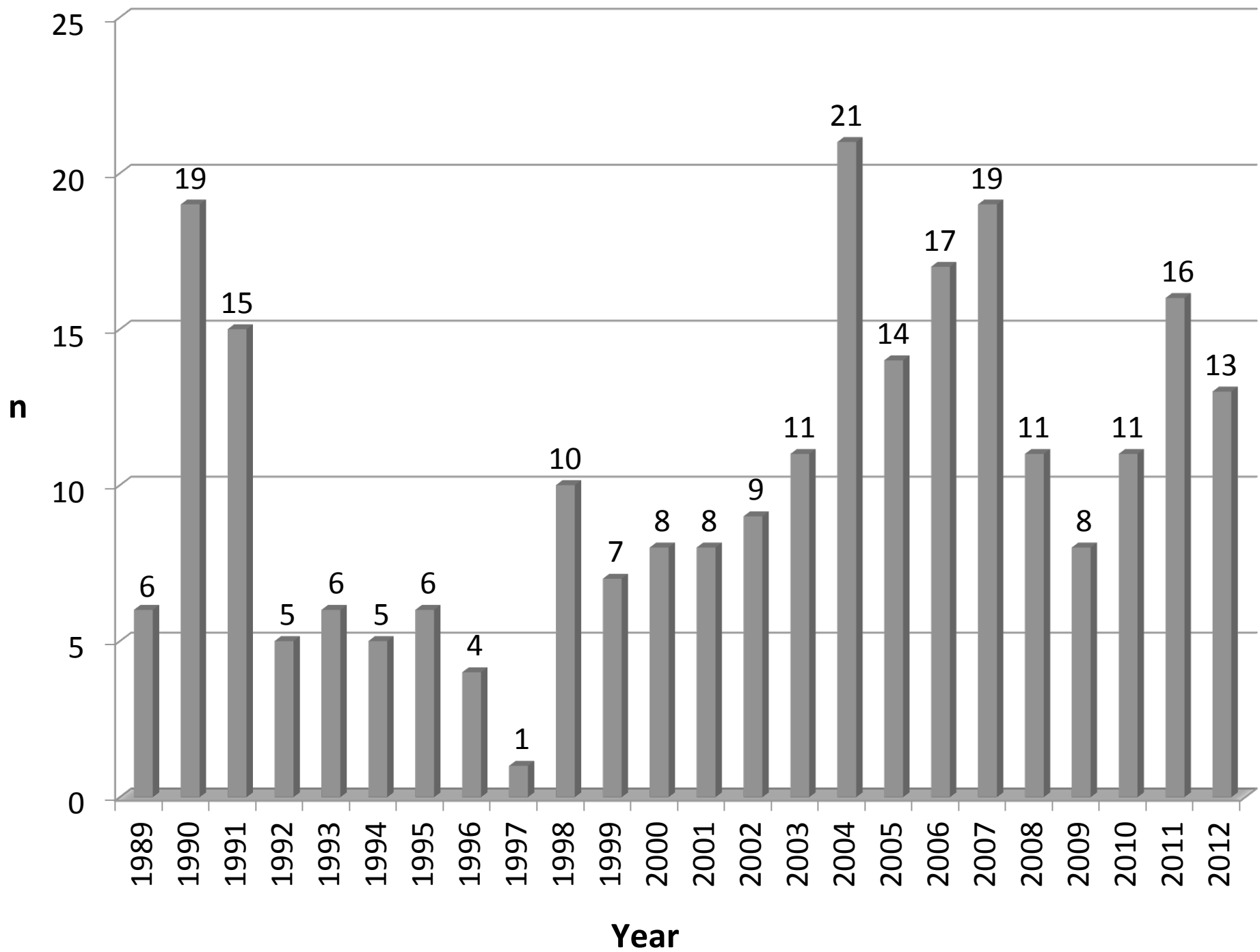

Supplement: Additional file 2: — Figure S2. Annual incidence. [file 40064_2015_1042_MOESM2_ESM.pdf]

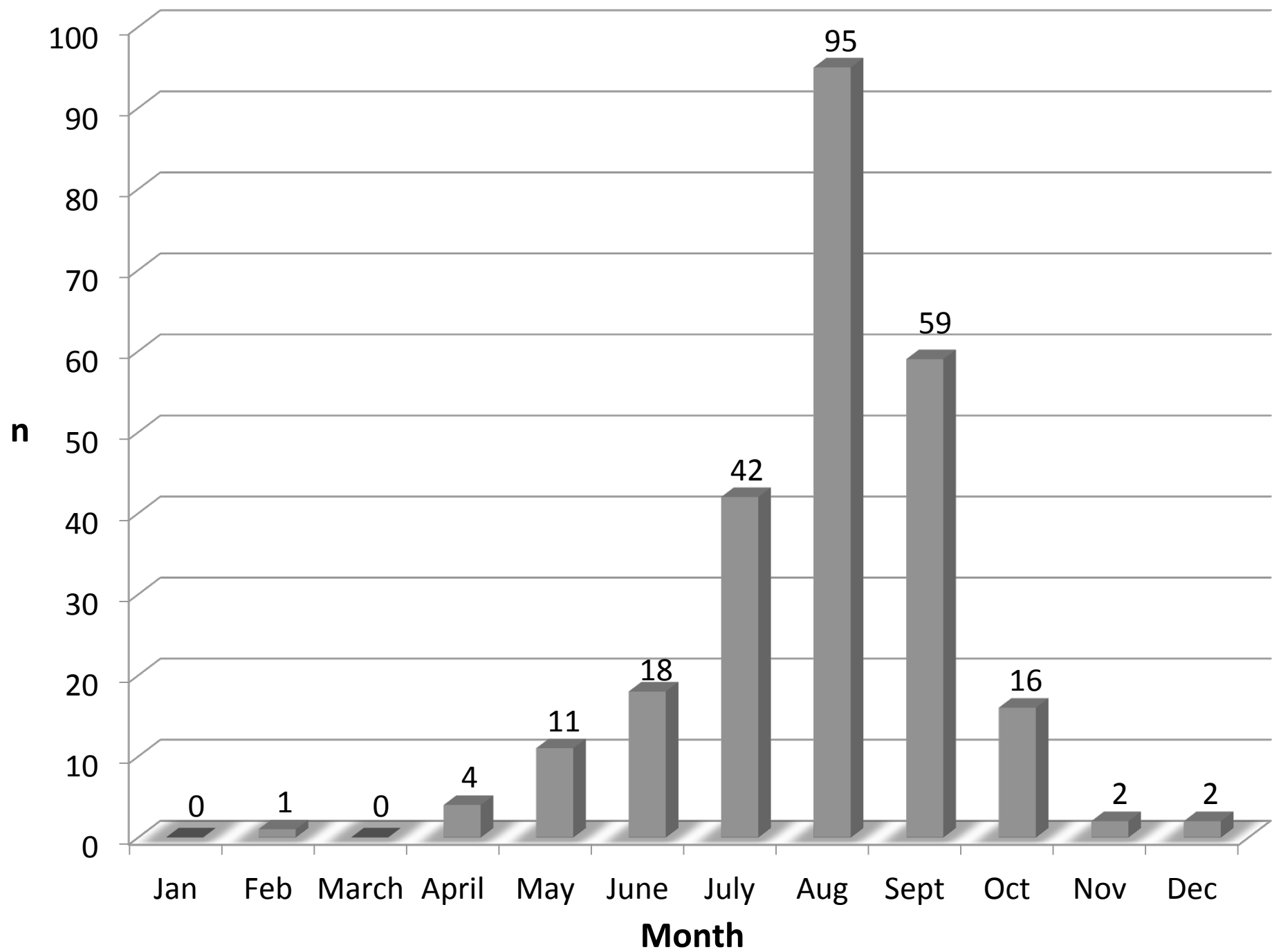

Supplement: Additional file 3: — Figure S3. Monthly distribution. [file 40064_2015_1042_MOESM3_ESM.pdf]
