# Supplementary material for: Mediterranean spotted fever: case series of 24 years (1989–2012)
Source: Springerplus. 2015 Jun 17;4:272. doi: 10.1186/s40064-015-1042-3 (PMC4469589; doi:10.1186/s40064-015-1042-3)
Supplement: Additional file 4: — Table S1. Most frequent comorbidities. [file 40064_2015_1042_MOESM4_ESM.docx]

**Table S1 – Most frequent comorbidities**

| **Pathology** | **n** | **%** |
| --- | --- | --- |
| Hypertension | 47 | 19 |
| Chronic Alcoholism | 31 | 12 |
| Diabetes | 18 | 7 |
| Chronic Obstructive Pulmonary Disease (COPD) | 18 | 7 |
| Heart failure | 12 | 5 |
