# Supplementary material for: Mediterranean spotted fever: case series of 24 years (1989–2012)
Source: Springerplus. 2015 Jun 17;4:272. doi: 10.1186/s40064-015-1042-3 (PMC4469589; doi:10.1186/s40064-015-1042-3)
Supplement: Additional file 5: — Table S2. Clinical manifestations. [file 40064_2015_1042_MOESM5_ESM.docx]

**Table S2 – Clinical Manifestations**

| **Clinical Manifestation** | **n** | **%** |
| --- | --- | --- |
| Fever | 245 | 98 |
| Exanthema | 218 | 87 |
| Myoarthralgia | 159 | 64 |
| Inoculation eschar | 151 | 60 |
| Headache | 119 | 48 |
| Asthenia | 68 | 27 |
| Altered mental status | 57 | 23 |
| Malaise | 54 | 22 |
| Shivers | 42 | 17 |
| Anorexia | 42 | 17 |
| Nausea and/ or vomiting | 41 | 16 |
| Sweating | 18 | 7 |
| Dyspnoea | 14 | 6 |
| Odynophagia | 13 | 5 |
| Agitation | 12 | 5 |
| Lipothymy | 10 | 4 |
| Dizziness | 8 | 3 |
| Abdominal pain | 7 | 3 |
| Altered balance | 4 | 2 |
| Convulsion | 3 | 1 |
| Hemiparesis | 2 | 1 |
