# Supplementary material for: Mediterranean spotted fever: case series of 24 years (1989–2012)
Source: Springerplus. 2015 Jun 17;4:272. doi: 10.1186/s40064-015-1042-3 (PMC4469589; doi:10.1186/s40064-015-1042-3)
Supplement: Additional file 6: — Table S3. Inoculation eschar location. [file 40064_2015_1042_MOESM6_ESM.docx]

**Table S3 – Inoculation eschar location**

| **Inoculation eschar location** | | **n** |
| --- | --- | --- |
| Not specified | | 2 |
| Head | | 4 |
| Trunk  (n = 87) | Axillary region | 12 |
|  | Shoulder | 14 |
|  | Thorax | 14 |
|  | Abdomen | 20 |
|  | Back | 8 |
|  | Buttock | 5 |
|  | Inguinal region | 10 |
|  | Genital | 4 |
| Upper Limbs  (n = 16) | Arm | 6 |
|  | Elbow | 2 |
|  | Forearm | 1 |
|  | Hand | 6 |
|  | Not specified | 1 |
| Lower Limbs  (n = 42) | Thigh | 23 |
|  | Knee | 3 |
|  | Leg | 8 |
|  | Foot | 5 |
|  | Not specified | 3 |
| Multiple (two) |  | 3 |
