# Supplementary material for: Mediterranean spotted fever: case series of 24 years (1989–2012)
Source: Springerplus. 2015 Jun 17;4:272. doi: 10.1186/s40064-015-1042-3 (PMC4469589; doi:10.1186/s40064-015-1042-3)
Supplement: Additional file 7: — Table S4. Serologic evaluation results (immunofluorescence assay). [file 40064_2015_1042_MOESM7_ESM.docx]

**Table S4 – Serologic evaluation results (immunofluorescence assay)**

| **1st Sample** | | **2nd Sample** | |  | **1st Sample** | | **2nd Sample** | |
| --- | --- | --- | --- | --- | --- | --- | --- | --- |
| **Titter** | **n** | **Titer** | **N** |  | **Titer** | **n** | **Titer** | **n** |
| **80** | 35 | **80** | 1 |  | **1280** | 9 | **640** | 1 |
|  |  | **160** | 5 |  |  |  | **1280** | 2 |
|  |  | **320** | 9 |  |  |  | **2560** | 1 |
|  |  | **1280** | 5 |  |  |  | **5120** | 1 |
|  |  | **2560** | 3 |  |  |  | **Not done** | 4 |
|  |  | **5120** | 1 |  | **2560** | 1 | **Not done** | 1 |
|  |  | **Negative** | 1 |  | **5120** | 1 | **Not done** | 1 |
|  |  | **Not done** | 10 |  | **Negative** | 134 | **80** | 16 |
| **160** | 14 | **160** | 3 |  |  |  | **160** | 11 |
|  |  | **320** | 1 |  |  |  | **320** | 16 |
|  |  | **640** | 1 |  |  |  | **640** | 12 |
|  |  | **1280** | 1 |  |  |  | **1280** | 8 |
|  |  | **Not done** | 8 |  |  |  | **2560** | 8 |
| **320** | 12 | **640** | 3 |  |  |  | **5120** | 1 |
|  |  | **1280** | 4 |  |  |  | **Negative** | 13 |
|  |  | **Not done** | 5 |  |  |  | **Not done** | 49 |
| **640** | 6 | **320** | 2 |  | **Not done** | 38 | **640** | 2 |
|  |  | **5120** | 1 |  |  |  | **Negative** | 1 |
|  |  | **Not done** | 3 |  |  |  | **Not done** | 35 |
